# Epilepsy Following Neonatal Seizures Symptomatic Of Stroke

**DOI:** 10.15844/pedneurbriefs-29-6-4

**Published:** 2015-06

**Authors:** Charu Venkatesan

**Affiliations:** 1 Division of Neurology, Department of Pediatrics, Cincinnati Children's Hospital Medical Center, Cincinnati, OH

**Keywords:** Stroke, Epilepsy, Neonates

## Abstract

Investigators from Child Neurology and Clinical Neurophysiology, Pediatric University Hospital, Padua, Italy studied the long term risk of developing epilepsy in patients with EEG confirmed neonatal seizures and arterial ischemic stroke.

Investigators from Child Neurology and Clinical Neurophysiology, Pediatric University Hospital, Padua, Italy studied the long term risk of developing epilepsy in patients with EEG confirmed neonatal seizures and arterial ischemic stroke.

This was a retrospective study where patients were recruited from a multi-center prospective registry. Patients with EEG confirmed seizures, arterial ischemic stroke confirmed by neuroimaging and follow-up ≥ 3.5 years were included.

55 patients from 10 centers were selected for the study. Mean gestational age was 40 weeks; 56% were males. The most frequent vascular territory involved was that of the left middle cerebral artery (49%), followed by the right middle cerebral artery (24%). Neonatal seizures occurred within the first week of life in all but 2 infants. 45% of infants had status epilepticus. Phenobarbital was an effective first-line medication in 56% of patients, while 36% needed more than one anti-epileptic drug. Phenobarbital was stopped prior to discharge in all patients. Mean follow-up was 8 years and 5 months. Development of epilepsy was noted in 16.4% of children at a mean age of 4 years 2 months. The risk of developing epilepsy was higher with involvement of the right middle cerebral artery and multiple arterial territories. [1]

COMMENTARY. Neonatal seizures occur in 1 per 1000 live births [2]. Etiology of neonatal seizures is diverse and includes vascular injury and metabolic and genetic disorders. Previous studies have reported higher rates of epilepsy following neonatal seizures. For example, a study by Clancy and Legido showed that 56% of neonates with seizures develop epilepsy; however, their study included patients with diverse etiologies of intracranial injury [3]. There is increasing awareness of the importance of examining the etiology of seizures in predicting outcome with respect to developing epilepsy. A recent study evaluating epilepsy after hypoxic ischemic injury in selectively head cooled infants found 18% of infants developed epilepsy and higher risk of epilepsy was associated with injury involving subcortical regions [4].

This study finds 16.4% of infants with neonatal seizures and perinatal arterial ischemic stroke developed epilepsy [1]. A retrospective study by Golomb et al., found that 67% of infants with perinatal arterial stroke developed epilepsy after 6 months of age; however, epilepsy resolved in a significant subset of patients and 25% of children with epilepsy continued to have epilepsy in longer term follow-up. Age at follow-up ranged from 9 – 179 months [5]. A retrospective study by Wusthoff et al. followed patients for a mean duration 31.3 months and found that the probability of remaining seizure-free at 3 years was 73% [6].

These studies suggest that the risk of developing epilepsy following perinatal arterial stroke is lower than previously thought. Future studies that continue to identify risk factors for development of epilepsy will allow us to implement improved vigilance, guidance and potentially preventive treatment. There are currently no guidelines on discontinuing anti-epileptic medications in neonates with perinatal arterial stroke who present with seizures. Accumulating data suggest that prolonged prophylactic therapy may not be needed in neonates.
